# Memorial Meeting Held by the Medical Society of the County of Erie

**Published:** 1902-09

**Authors:** Franklin C. Gram


### Memorial Meeting Held by the Medical Society of the County of Erie.

REPORTED BY FRANKLIN C. GRAM, M. D., Secretary.

### DR. THOMAS LOTHROP.

PURSUANT to a call of the President the Medical Society of the County of Erie, held a special meeting in the parlors of the Y.M.C.A., Pearl and Mohawk Streets, at 5 p.m., August 9, 1902, in memory of Dr. Thomas Lothrop, who died at his residence in Buffalo, August 7, 1902, aged 66 years 3 months and 22 days.

In the unavoidable absence of the president and the vice-president, the secretary, Dr. Franklin C. Gram, called the meeting to order and, on motion of Dr. Hopkins, Dr. Conrad Diehl was elected chairman, *pro tempore*.

Dr. DIEHL briefly stated the object of the meeting, and in the course of his feeling remarks said Dr. Lothrop was a former president of this society and had, for many years, occupied a prominent position in the medical profession as well as in the community and it was befitting the occasion that so many representative physicians were in attendance. He had known Dr. Lothrop for about forty years and respected him as a man of good judgment and high professional attainments.

Dr. H. R. HOPKINS moved that a committee of five be appointed to prepare a suitable memorial and present it to the meeting for approval. The motion was adopted and the chair appointed as such committee Drs. Henry Reed Hopkins, John Hauenstein, William Warren Potter, Devillo W. Harrington and Joseph Fowler.

The committee withdrew and soon presented the following:

### In Memoriam.

THOMAS LOTHROP, M.D., 1836-1902.

Forasmuch as it hath pleased Almighty God, in His wise providence, to take out of this world the soul of our deceased brother, Dr. Thomas Lothrop, we, the Medical Society of the County of Erie, therefore place upon record our testimony and our appreciation of the noble qualities of mind, character and person which distinguished in a singular and remarkable degree his professional career.

And chiefly are we bound to praise his steadfast loyalty and devotion to the highest ideals of professional and personal life and conduct. He was ever a loyal friend, an open champion, an ardent advocate of all that was purest, most elevating, most ennobling in the profession of medicine which he ever adorned, and which in turn rewarded him with its chiefest honors.

Also, we gladly testify that in like manner as his professional ideals were high and dignified, so were his sympathies and charities broad and discriminating; having a lively sense of the uplifting power of education, he constantly exercised himself in affording to others the opportunities of self-development, the high possibilities of which his own person and career so well illustrated.

Doctor Lothrop was, as a citizen, courageous and inflexible in support of municipal progress, as a physician successful in an unusual degree not only in attracting and retaining the confidence of a large and wealthy patronage, but also in winning and holding the confidence of his colleagues, by his wise, conservative, yet progressive practice; as a man an illustrious example of the genial, chivalrous christian gentleman. May light perpetual shine upon him!

HENRY REED HOPKINS,<br>

JOHN HAUENSTEIN,<br>

WILLIAM WARREN POTTER, } Committee.<br>

DEVILLO W. HARRINGTON,<br>

JOSEPH FOWLER,

THE MEDICAL SOCIETY OF THE COUNTY OF ERIE,<br>
BUFFALO, August 9, 1902.

In moving the adoption of the memorial Dr. Hopkins added a brief personal tribute. He also said that it had long been contended that a physician must not deal with anything but medicine. Dr. Lothrop since early life did not hesitate to take his stand as a citizen. He soon demonstrated that he measured up to the requirements and showed that a professional training aptly fits a man for the duties of citizenship.

Dr. WILLIAM WARREN POTTER, in seconding the motion of Dr. Hopkins, stated that it was his privilege to enjoy Dr. Lothrop's acquaintance for more than twenty-five years, and he could add little to the memorial which succinctly presents some of his principal characteristics. Dr. Lothrop was a professional example worthy of emulation. He stood for everything ennobling. For a period of ten years, the speaker was associated with Dr. Lothrop in the conduct of the medical journal and thus learned to know him in a more intimate relation than otherwise would have been the case. Dr. Lothrop always advocated a strong policy and insisted on a high standard.

The example of such a man was something to remember, something to revere, something to follow.

Dr. J. Henry Dowd gave some reminiscences of the period when Dr. Lothrop occupied the chair as professor of obstetrics at Niagara University. He spoke of his commonsense lectures and his practical methods.

Dr. J. B. Coakley alluded to his work as director of the State Hospital, his thirty years connection with the Church Home, and some of his many public and private charities.

Dr. H. D. Ingraham became acquainted with Dr. Lothrop immediately after coming to Buffalo. In addition to what had already been mentioned the deceased was a great friend of poor students and young practitioners, some of whom were educated at Dr. Lothrop's personal expense.

Dr. D. W. Harrington, alluding to his strong character, said if Dr. Lothrop was your friend you knew it; if your enemy, you would have no doubts on the subject. He was a man of integrity and truthfulness.

Dr. A. A. Hubbell said he had been associated with Dr. Lothrop for many years. The doctor was a self-made man, not only in medicine but in other accomplishments. He stood for the highest and best, was uncompromising in establishing and maintaining the highest standard for Niagara Medical College. He had excellent business qualities, and arranged the affairs of his department with such foresight that the maternity which he established proved a benefit to the college and the community, as well as a profitable investment.

Dr. W. G. Taylor said he was the young practitioner's best friend and always straightened out the most difficult complications without compromising anybody.

Dr. P. W. Van Peyma, remarked among other things, that he was one of the first associates of Dr. Lothrop in the proprietorship of the Journal, and although they sometimes disagreed on questions of policy, yet they always remained friends. He was a man for whom he had the highest respect.

The chair then put the motion on the adoption of the memorial, and it was carried without division.

On motion of Dr. Callanan it was ordered that the society attend the funeral in a body, and then the meeting adjourned.

